# Salt tolerance QTLs of an endemic rice landrace, *Horkuch* at seedling and reproductive stages

**DOI:** 10.1038/s41598-022-21737-9

**Published:** 2022-10-15

**Authors:** Taslima Haque, Sabrina M. Elias, Samsad Razzaque, Sudip Biswas, Sumaiya Farah Khan, G. M. Nurnabi Azad Jewel, Md. Sazzadur Rahman, Thomas E. Juenger, Zeba I. Seraj

**Affiliations:** 1 Plant Biotechnology Lab, Department of Biochemistry and Molecular Biology, University of Dhaka, Dhaka, 1000 Bangladesh; 2 Department of Integrative Biology, University of Texas, Austin, TX 78712 USA; 3 Department of Agronomy and Horticulture, University of Nebraska, Lincoln, NE 68583 USA; 4 School of Life Science, Independent University, Dhaka, 1229 Bangladesh; 5 Department of Soil and Crop Sciences, Texas A&M University, College Station, TX 77843 USA; 6 Department of Biochemistry and Molecular Biology, Jagannath University, Dhaka, 1100 Bangladesh; 7 Department of Genetic Engineering and Biotechnology, Shahjalal University of Science & Technology, Sylhet, Bangladesh; 8 Plant Physiology Division, Bangladesh Rice Research Institute, Gazipur, Bangladesh

**Keywords:** Genetics, Plant sciences

## Abstract

Salinity has a significant negative impact on production of rice. To cope with the increased soil salinity due to climate change, we need to develop salt tolerant rice varieties that can maintain their high yield. Rice landraces indigenous to coastal Bangladesh can be a great resource to study the genetic basis of salt adaptation. In this study, we implemented a QTL analysis framework with a reciprocal mapping population developed from a salt tolerant landrace *Horkuch* and a high yielding rice variety *IR29*. Our aim was to detect genetic loci that contributes to the salt adaptive responses of the two different developmental stages of rice which are very sensitive to salinity stress. We identified 14 QTLs for 9 traits and found that most are unique to specific developmental stages. In addition, we detected a significant effect of the cytoplasmic genome on the QTL model for some traits such as leaf total potassium and filled grain weight. This underscores the importance of considering cytoplasm-nuclear interaction for breeding programs. Finally, we identified QTLs co-localization for multiple traits that highlights the possible constraint of multiple QTL selection for breeding programs due to different contributions of a donor allele for different traits.

## Introduction

Rice (*Oryza sativa* L.) production, which feeds almost half of the world population, is under threat from global environmental changes such as increasing salinity, heat and drought^1,2^. Among these abiotic stresses, salinity has already affected 45 million hectares of irrigated land worldwide and 1.5 million additional hectares are impacted each year^3^. Bangladesh and other locales at or near sea level are particularly vulnerable to climate change-induced salinity. In Bangladesh, about 30% of the cultivable land along the coast is affected by salinity due to tidal flood during the wet season resulting in direct inundation by saline water, and upward or lateral movement of saline ground water during the dry season^4^.

High yielding “elite” rice cultivars are especially susceptible to salinity stress. Recent studies have shown that production of high yielding rice varieties in Bangladesh will decrease by 15.6% in coastal districts where soil salinity is predicted to exceed 4 deciSiemens per meter (dSm^−1^) by 2050^5^. However, the coastal belt of Bangladesh is enriched with many local rice landraces, among which a handful are adapted to high-to-moderate soil salinities. The rice landrace, *Pokkali*, has long been used as a salt tolerant landrace reference. Many other salt-tolerant landraces such as *Horkuch*, *Ashfal*, *Jatai* and *Balam* from the coastal region of southern Bangladesh have been identified and are currently grown by farmers in these salt-affected regions^6,7^. Unfortunately, these landraces suffer from low yield, poor grain quality and longer duration to reach maturity and therefore not suitable as commercial varieties. However, studies of these adapted landraces to understand their salt tolerant mechanisms can help to incorporate desired traits into commercial rice. Therefore, it is important for breeders to identify genetic variants of salt stress responses in these naturally adapted landraces in order to design highly salt tolerant elite rice.

The effect of salinity on rice growth varies across various developmental stages^8^. It is most sensitive to salinity at the early seedling stage and during panicle formation, and relatively tolerant during early germination, active tillering and maturity^8–13^. During salt stress at the early seedling stage, there is a significant decrease of dry matter as well as quantum yield of PSII and a significant increase of sodium concentration in root, stem and shoot tissue^14^. At the reproductive stage, salinity stress produces a significant decrease in panicle weight, panicle length, primary branches per panicle, filled grains per panicle, total seeds per panicle, total seed weight per panicle, 1000-seed weight and total seed weight per plant^15,16^. However, Moradi et al.^17^ have shown that salinity tolerance at the seedling and reproductive stages is only weakly associated. This emphasizes the importance of discovering the contributing traits of these two very important growth stages of rice.

The physiological basis of salt tolerance during the early seedling stage is well understood. Munns and Tester^3^ and Roy^18^ have proposed several physiological mechanisms of seedling tolerance such as sodium exclusion, compartmentalization of excessive sodium ions (tissue tolerance) and shoot-ion independent tolerance for early stage tolerance. It has been reported that *Pokkali* maintains lower shoot Na^+^ accumulation and lower shoot Na^+^/K^+^ ratio under high salinity compared to sensitive genotypes^19,20^. The enhancement of salinity tolerance by constitutive overexpression of the vacuolar Na^+^/H^+^ antiporter gene from *Pokkali* in transgenic rice plants suggest that this landrace may use a tissue tolerance mechanism to lower shoot Na^+^/K^+^ ratio under high salinity^21^. Negrao et al.^22^ genotyped 392 rice accessions by EcoTILLING in order to understand allelic difference for salt stress for different salt adaptive rice accession. Their study suggest that none of the main three mechanisms of tolerance is preferentially used over another^23^. Therefore, studies of different landraces can offer ways to understand individual salt tolerance mechanisms in the rice plant^6,24^. However, the mechanism associated with tolerance during the reproductive stage has been barely explored. As mentioned earlier, high salinity in this stage can alter many traits associated with grain quality and quantity, eventually decreasing yield significantly. Therefore, it is important to explore the physiological response of rice at both the stages in order to obtain a superior variety which can maintain salinity tolerance for both developmental stages.

The choice of female parents in breeding programs plays a critical role due to complex nuclear–cytoplasmic interactions that may alter phenotypes in both interspecific and intraspecific crosses. However, it still remains unclear to what extent these two components interact with each other and the role of environment in this interaction. Gregorio and Senadhira^25^ have studied the genetics of salinity tolerance with diallelic reciprocal crosses of nine different rice varieties and found significant reciprocal effects among many crosses. The presence of maternal inheritance has also been reported for other abiotic stresses such as chilling response^26^ and drought^27^. Therefore, plant breeding programs to produce stress tolerant high-yielding varieties, have to take into account a specific cytoplasm and its interactions with nuclear donor alleles for determining the performance of plants under stress.

Quantitative trait loci (QTL) mapping has been implemented in rice to explore the genetic basis of traits involved in salinity stress for seedling stages, including salt injury/tolerance score, fresh and dry weight of shoot and root, Na^+^ and K^+^ content of shoot and root, and chlorophyll content^28–36^. Several potassium transporters and plasma membrane Na^+^/H^+^ exchanger such as HKT^37^, SOS1^38^ have been reported as candidate genes which are involved in the leaf ion homeostasis during salinity stress. For instance, fine mapping of one such QTLs further identified *SKC1* as a candidate transporter which mediated K^+^ homeostasis in the salt-tolerant variety under salt stress at seedling stage^29,30^. However, very few studies have been conducted to understand the genetic basis of reproductive stage traits that are important to confer tolerance such as plant height, tiller number, panicle number, pollen fertility and yield^39,40^.

QTL co-localization has been reported for traits that are strongly correlated^41^. Many clustered, putatively pleiotropic QTL have been found that affect various life history and fitness characters, especially those that are related to yield, in rice, wheat, pea and rapeseed^42–45^. QTL for two different traits can have the same/opposite sign of effects. For breeding programs that aim to pyramid QTL for multiple desired traits, oppositely signed QTL for different traits may impose some constraints on selecting co-localized QTL. Hence, it is beneficial to have an explicit understanding of the sign and effect size of  co-localized QTL and perform careful selection of these genomic loci for pyramiding.

In this study, we genotyped a reciprocal mapping population developed from a cross of the salt tolerant landrace, *Horkuch* and a high yielding variety *IR29* by a DArTseq technique^46^, extending earlier work using ddRAD genotyping^47^. Using an improved genetic map and more sophisticated QTL mapping methods, we identified 14 QTLs for 9 traits for salinity treatments at two different developmental stages of the rice plant. One important finding of this study was that the cytoplasm donated from the maternal parent played an important role in a plant’s performance under salinity stress. We identified co-localized QTLs within and across two different developmental stages, which emphasizes the need for conditional selection of QTL in breeding programs in order to combine survivability at seedling stage and yield maintenance at reproductive stage. Taken together, the findings of this study contribute to our understanding of the molecular mechanism of salt tolerance for *Horkuch* and pave a way to introgress salinity tolerance into a commercial cultivar that can maintain significant yields under stress.

## Materials and methods

### Development of the reciprocally crossed populations and physiological screening

This study complied with relevant institutional, national, and international guidelines and legislation for handling plant materials. The rice cultivars *Horkuch* (IRGC 31804) and *IR29* (IRGC 30412) were used as parents in a cross to generate a bi-parental reciprocal mapping population. The materials were collected from the IRRI rice Genbank using government issued relevant import permits. Details of the methods for developing this mapping population and its physiological screening can be found in Elias et al.^48^ During wet season of 2011 (June July) the parents were sown at International Rice Research Institute (IRRI) crossing block. The F_1_ hybrids were confirmed by RM493 SSR marker. The plants were advanced to F_2_ generation using single seed descendent method and F_3_ population was generated by selfing F_2_ population. In brief, our experimental approach centers on an F_2:3_ design, whereby genotypes for mapping are collected from F_2_ individuals and phenotypes are obtained from a sampling of their F_2:3_ progenies^46^. In this work, F_2:3_ progenies derived from *Horkuch* (mother) × *IR29* (father) will be referred to as *Horkuch*♀ and those from *IR29* (mother) × *Horkuch* (father) as *IR29*♀. We randomly chose 137 families from the *IR29*♀ set and 65 families from *Horkuch*♀ set for seedling stage QTL analysis. Three replicates seedlings from each family were grown in hydroponic system containing Yoshida culture solution^49^ following a balanced incomplete block design. Salinity stress of 6 dS/m was applied on 13th day as a supplement with Yoshida solution and carried out for 4 days by 2 dS/m increment per day. We measured Standard Evaluation system (SES), Shoot Relative Water Content (SRWC), Shoot Length (SL), Root Length (RL), Total Chlorophyll Content (Tchlr), Total Sodium (TNa) and Total Potassium (TK) at the end of salinity treatment. Detailed method for plant growth and phenotyping can be found in Elias et al.^48^. In order to handle bulk volume of mature plants efficiently, for the reproductive stage QTL analysis, only 140 families were chosen based on a selection of 70 F_2_ families from each population. Our selection was based on the distribution of SES)scores where the lower tail (more tolerant families) was defined as SES scores from 3 to 5 and the upper tail (sensitive) was defined as SES scores from 7 to 9. All families irrespective of the maternal inheritance that were in the lower or upper tail were selected along with 70 randomly chosen families from SES score in between 5 and 7. The two parents were also included in our studies. A few families had poor germination and were subsequently excluded from the reproductive screening experiment. In the end, 130 families were included in reproductive screening: 61 from *Horkuch*♀ and 69 from *IR29*♀ population. Plants were grown in perforated plastic buckets filled with processed field soil and kept within a large plastic bowl filled with water. Salinity treatment was carried out as described by Gregorio et al.^50^*.* We measured Days to flowering (DF), Plant Height (PH), Total number of Tiller (TT), number of Effective Tiller (ET), Panicle Exsertion (PE), Filled Grain Number (FWN), Filled Grain Weight (FGW), Spikelet Fertility (SF), and Harvest Index (HI).

### DNA extraction, genotyping by DArTseq and linkage map construction

Genomic DNA of F_2_ individuals and parents was extracted using the CTAB method (Doyle and Doyle, 1990) from 1 g of fresh leaf tissue after freezing in liquid nitrogen and grinding. Genotyping was done by the DArTseq technique as described by Akbari et al.^46^. For the DArTseq method Nipponbare Genome from Phytozome (v9) was used as reference to determine the physical position of each DArTseq clone. We filtered the DArTseq Single Nucleotide Polymorphisms (SNPs) and retained only loci that: (a) are homozygous for both the parents and (b) are polymorphic between parents. Analyses for linkage map construction were completed with qtlTools^51^ and R/qtl^52^ packages. We filtered markers that had more than 50% missing data and showed significant segregation distortion based on a chi-square test (p-value < 0.001) from expected ratio (For a given locus, 1:2:1 ≡ homozygous parent 1 allele: heterozygous: homozygous for parent 2 allele). Similar markers were further removed using the dropSimilarMarkers function of qtlTools package^51^ in R with a minimum recombination fraction threshold of 0.03. Marker order was obtained by the tspOrder function from TSPmap tool^53^ which applies a traveling salesperson problem solver to order markers using Hamiltonian circuit. Markers which had discordance (have very different orders in genetic map vs. physical map) were also removed. Before estimating the linkage map a few alleles that showed erroneous calls were masked manually. The final linkage map was estimated with the est.map function of R/qtl package with the Kosambi map function using an error probability threshold of 0.001. Hybrid incompatibility may attribute for the nonrandom association of parental nuclear alleles with respective parental cytoplasmic background. To test for the occurrence of cytoplasm-nuclear genetic association, we performed chi-square test of independence on allele frequency for each locus grouped by cytoplasm. For a given locus the null expectation was no difference in the ratio of allele frequencies among two different cytoplasmic background. Significant association was determined by adjusting p-values using False discovery rate (FDR) methods (FDR threshold = 0.1).

### QTL analysis

Genotype probabilities were calculated at a 1 cM step interval using the calc.genoprob function. QTL mapping was executed using the Haley–Knott regression algorithm of the R/qtl2^54^ package. We fit the LOCO (Leave One Chromosome Out) model for each trait and included kinship as a covariate. The LOCO model utilizes the kinship matrix to reduce background polygenic variation except for the chromosome that is being tested for QTL mapping and minimize the over estimation of QTL effect size. We also tested cytoplasm as an additive and interactive covariate in the QTL model using likelihood ratio tests and retained factors in the models when significant. Following is a general model formula for QTL identification of a single trait:

$${\text{Y }}\left( {{\text{trait}}} \right) \, = \, \mu \, + {\text{ QTL }} + {\text{ cytoplasm }}\left( {{\text{if}}\,{\text{appropriate}}} \right) \, + {\text{ QTL}} \times {\text{cytoplasm }}\left( {{\text{if}}\,{\text{appropriate}}} \right) \, + kinship \, + \, error$$

To test whether our selection of cohorts imposed significant population structure for the reproductive stage treatment we assigned each F_2_ family into one of the following categories: tolerant, intermediate and sensitive. We incorporated selection cohort as a covariate while building the primary QTL model for traits at reproductive stage. However, we did not find any significant effects of selection cohort on QTL models and therefore did not include selection cohort as a covariate for further analysis. Significance thresholds for QTL were determined for each trait by 1000 permutations (alpha = 0.05) and QTL peaks that passed the threshold were considered for further analysis. Permutations were stratified by cytoplasm for the QTL models where cytoplasm was considered as a covariate. We also evaluated the normality of the QTL model residuals. Confidence intervals (1.5 LOD drop) for each QTL were calculated using the *lodind* function of the R/qtl package expanding it to a true marker on both sides of the QTL. Percent variance explained by each QTL for a given trait was estimated by the *fitqtl* function from the R/qtl package. Mean and standard error of grouped phenotype for different genotype x cytoplasm combinations for the marker at QTL peak was estimated by *effectplot* function of the R/qtl package. Codes for QTL analysis are available in GitHub repository (https://github.com/tahia/RiceSaltQTLAnalysis).

### Identification of candidate genes within QTL confidence interval and gene ontology enrichment analysis

In order to identify candidate gene models within a given QTL interval, we integrated the genetic and physical maps based on the marker order of the genetic map. We first pulled out the genetic markers flanking a given QTL confidence interval and their basepair positions to define the physical interval on the genome for that QTL. Gene models in these physical QTL intervals were retrieved using the structural gene annotation of the rice Nipponbare reference genome from Phytozome 9. We used the Gene ontology (GO) annotated for each gene model of this reference for GO enrichment analysis. We then tested for the enrichment of GO terms for each QTL interval using the classical Fisher’s exact test available in the topGO^55^ package in R.

## Results

### Phenotypic traits vary between cross direction in both developmental stages

In our previous study, we used this bi-directional F_2:3_ population to examine the effect of salinity on various growth, yield and physiological parameters of rice as well as the role of cytoplasm on these traits and reported that maternal inheritance contributed to salt tolerance for F_3_ progenies^48^. Rice is most susceptible to salinity during seedling and reproductive growth stages. In the current study, we focused on a subset of phenotypes from Elias et al.^48^ that can potentially mediate stress during salinity treatment at these two developmental stages and more directly assess the effect of cytoplasm-nuclear interaction leveraging this bi-directional mapping population. For the seedling stage, we evaluated traits that were related to survival, photosynthesis and mineral elements in leaves and for the reproductive stage treatment, we focused on yield-related parameters.

**Figure 1:**
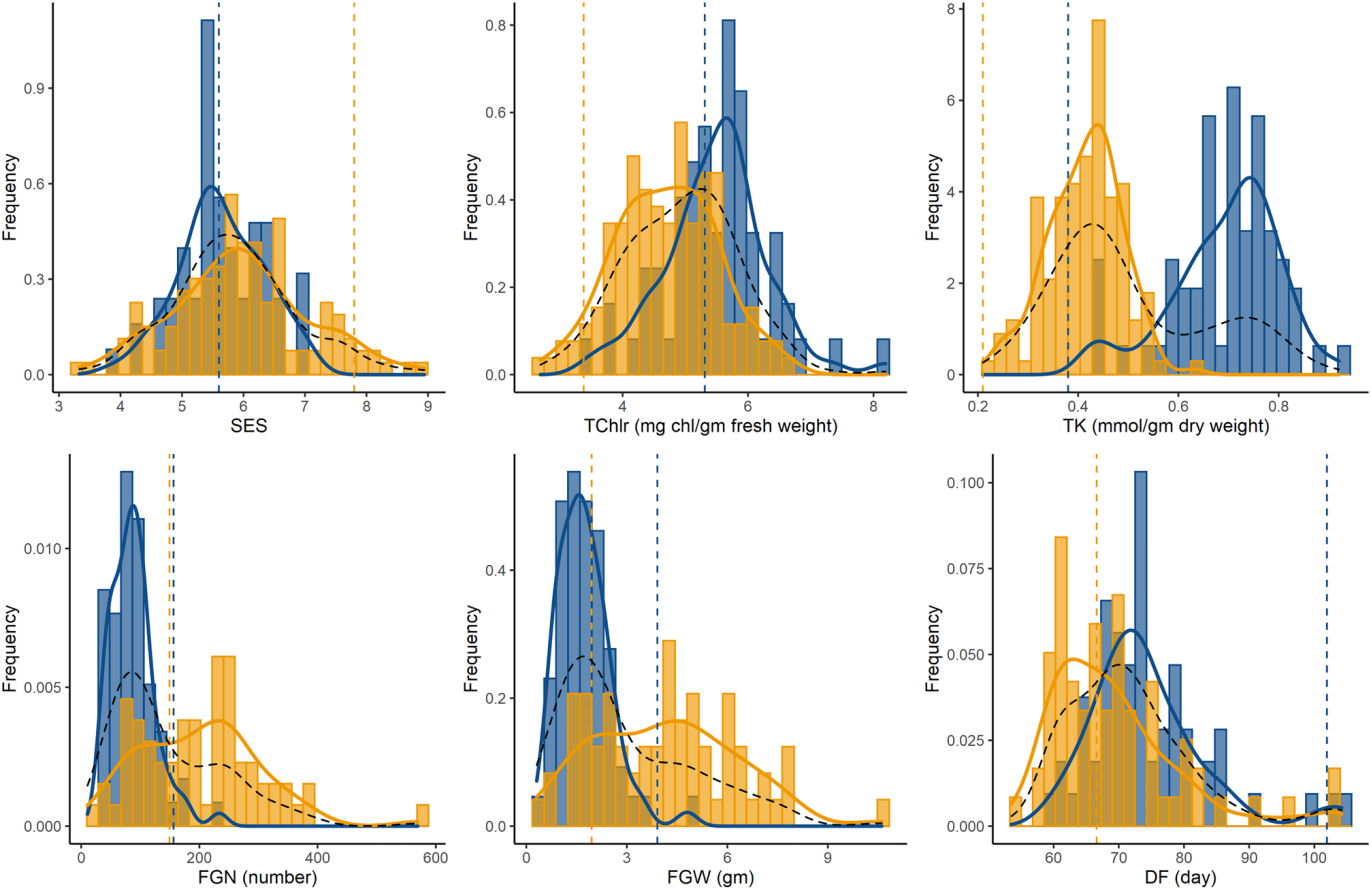
Frequency distribution of traits showing transgressive segregation in the F_2_ population and the individual subsets of cross directions. Blue and orange histograms indicate samples from *Horkuch*♀ and *IR29*♀ cytoplasm respectively. Curves in blue and orange indicate distribution plots of *Horkuch*♀ and *IR29*♀ cytoplasm respectively and dotted curve in black indicates the distribution plot of total population. Parental values are marked by a dotted vertical line where blue indicates *Horkuch* and orange indicates *IR29*.

We found a striking difference between the two parents for many of our measured traits across both stages of salt treatment (Supplementary Table S1). As reported in previous studies, we found that *Horkuch* is more tolerant to salinity (measured by SES score) compared to *IR29* which is highly sensitive for salt at the seedling stage (p-value < 0.01). For reproductive stage salinity treatment, these two parents show significantly different responses for ET, SF, FGW, DF and HI where the *Horkuch* parent had higher ET, SF, FGW and HI. For progenies, we found many traits were significantly different as a result of cytoplasmic background (Supplementary Table S1, Fig. 1). TChlr, TNa, TK and K/Na were found to differ significantly among the *Horkuch*♀ and *IR29*♀ at the seedling stage treatment. For reproductive stage treatment PE, TT, ET, FGN were found to differ significantly between these two parental cytoplasms. This observation indicates that cytoplasm can explain a significant amount of variation in the population.

Subsequently, we investigated the role of cytoplasm on the transgressive segregation of these traits. In this experiment we found that SES, SRWC, TChlr, TK, FGN, FGW clearly segregate transgressively (Supplementary Table S1). Interestingly, TK, TChlr, FGN and FGW showed significant differences in the two reciprocal crosses and demonstrated a biomodal distribution with respect to their maternal background. The distribution of TK showed a strong bimodal pattern with very little overlap of distribution between cytoplasmic backgrounds (Fig. 1). This observation is indicative of an important role of cytoplasm on transgressive segregation for this population.

**Figure 2:**
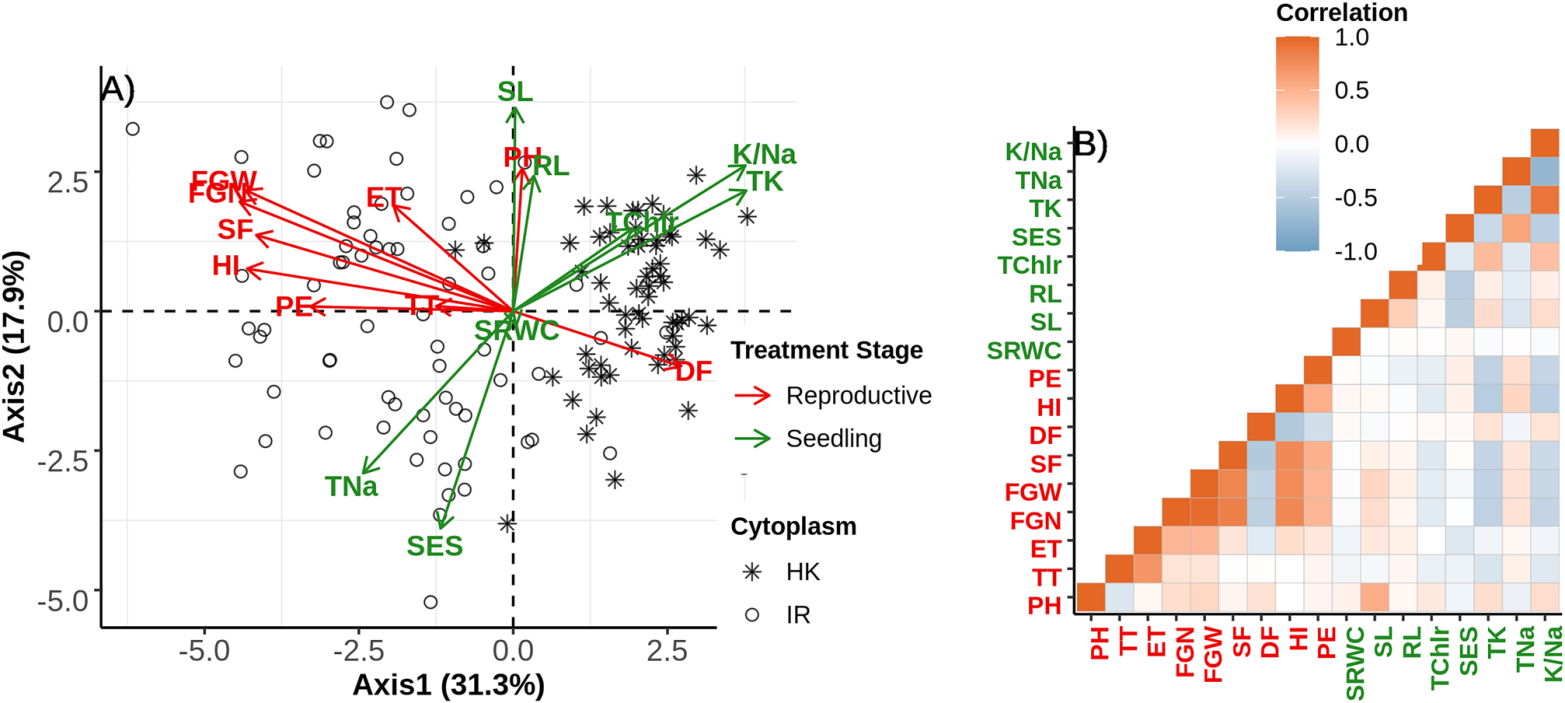
(**A**) PCA on trait correlations in the F_2_ mapping population. Each point represents the genetic means of each F_2_ family whereas the shape of point indicates the cytoplasm (cross direction). Direction of variation for axis 1 and 2 of each trait has been plotted as arrow and are color labelled depending on two different treatment stages: green indicates Seedling stage treatment and red indicates reproductive stage treatment. Labels of traits are printed close to the arrow-head. (**B**) Plot shows the correlations of traits where brown color shows positive correlation and light-blue indicates negative correlation. Traits labelled with green color indicates seedling stage ones and red indicates reproductive stage traits.

To understand the partitioning of genetic variation, we used principal component analysis of 130 families which had complete observations of all traits for both the stages of treatment. The first two PC axes comprise the majority of genetic trait variation in this population (31.3%) and 17.9% respectively (Fig. 2). The first principal component separates individuals into two groups almost exclusively depending on their respective cytoplasm. This observation further supports a significant role of cytoplasm on the performance of a plant under different treatment stages. We also found a moderate correlation of some traits between these two different stages of treatment: PH showed positive correlation with SL and yield related traits such as FGN, FGW, SF, HI and PE is negatively correlated with TK (Fig. 2). This correlation suggests some possible shared mechanisms of salinity responses which trades off between two different treatment stages.

### Linkage map construction

In our previous study we constructed a linkage map for another subset of this population by applying a ddRAD technique^47^. Unfortunately, we had high genotyping error and inflated segregation distortion for many loci for the two parental alleles for a given locus. As a result, we failed to effectively cover the genomic space, including poor sample of the entire space represented by chromosome 5. In this study, we genotyped using Diversity Array Technology coupled with Sequencing (DArTSeq) technique in order to reduce genotyping error and genome representation by adding higher sequence coverage. DArTSeq can generate low to moderate density SNP information with high coverage and low cost. This method uses restriction enzymes to reduce the complexity of the genome and has been optimized for various plant species to achieve optimal complexity reduction. We used this platform for our mapping population to generate genotype information at ~ 10 thousand loci that are well-distributed in the rice genome. We obtained 2230 high quality SNPs that are polymorphic homozygous SNPs for the parents and has < 50% missing data for population. Subsequently, we dropped markers which demonstrated significant segregation distortion or very low recombination and obtained a final map of 499 markers with a map size of 2004.8 cM and average inter markers distance of 4.1 cM (Supplementary Fig. S1A). The maximum gap of 22.3 cM was found in chromosome 5. Chromosome 7 had the fewest markers (22 markers). Supplementary Figure S1B presents the concordance of genetic map with the physical map of rice genome. As mentioned earlier, we detected significant association of cytoplasm for multiple traits in both stages of treatment and therefore we tested (Chi-square test of independence, see “Methods” section) for cytoplasm-nuclear association for each marker in this genetic map. We detected 10 loci that showed significant cytoplasm-nuclear association [FDR < 0.1] and these are mostly clustered in chromosomes 2, 3 and 7. This association suggested the presence of non-random cytoplasm-nuclear allelic assortment in this mapping population resulting from selection at the reproductive stage during the development of the mapping population. Overall, we constructed a linkage map with moderate marker density that was closely aligned with the physical map of the rice genome.

### Significant QTL at both growth stages

#### QTL for seedling stage treatment

**Figure 3:**
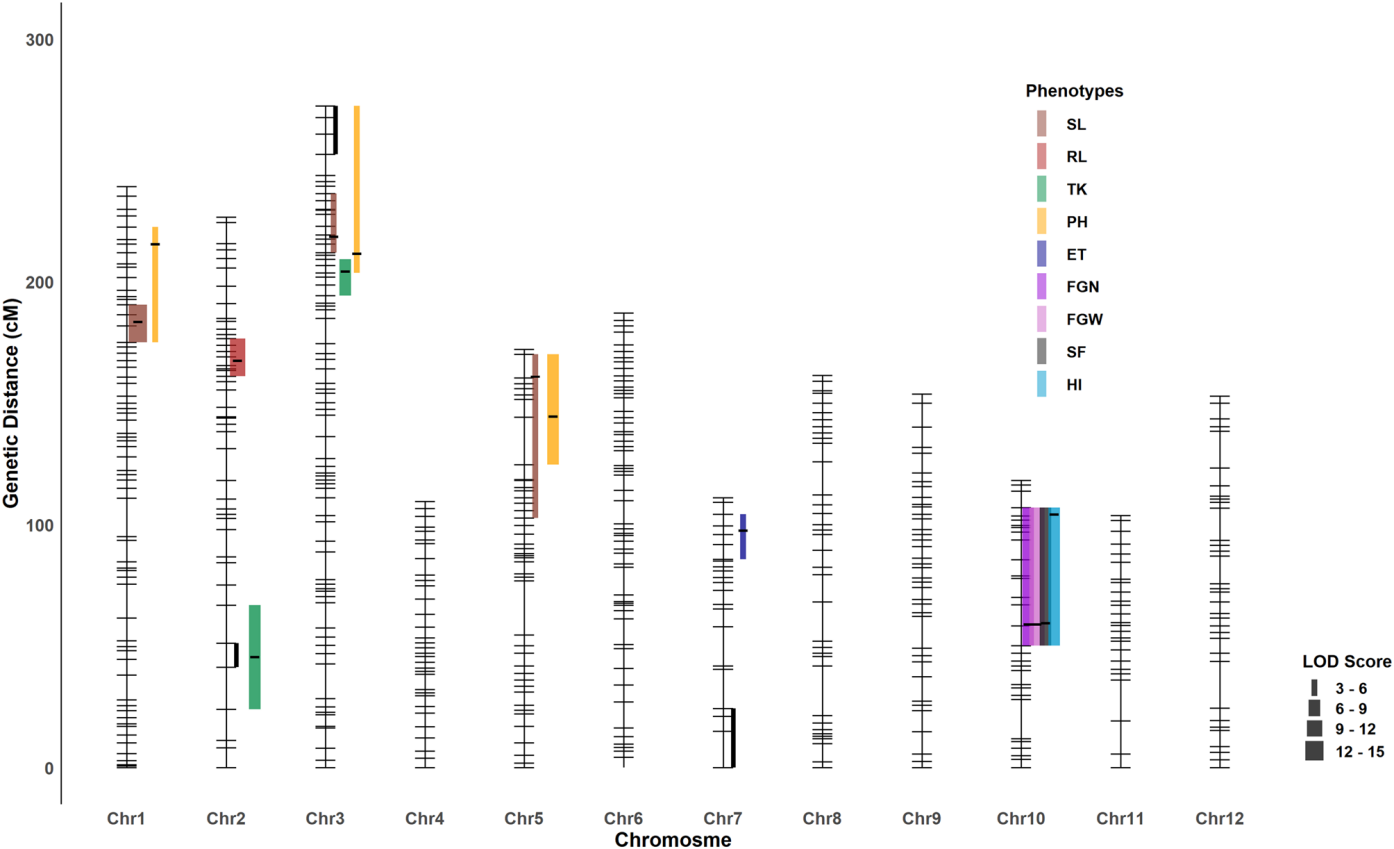
Illustration of QTL across chromosomes. QTL are denoted as a point and 1.5 LOD drop confidence intervals extended to a true marker is indicted by the bar for each QTL. Peaks of the QTL were marked as black line in QTL intervals. QTL from same trait are marked with same color. Line width represents the magnitude of LOD score. Genomic regions that showed significant association with cytoplasm are marked here with black line segment.

We measured eight traits that reflect the survival performance of rice seedlings under salinity stress. We detected six QTLs for three traits: SL, RL and TK (Table 1, Fig. 3). We found three significant QTLs for SL occurring at qSL.1@183 (reporting a QTL for SL located at chromosome 1 at 183 cM), qSL.3@218, and qSL.5@160. For these QTLs, the positive alleles were from *Horkuch* parent and we detected no significant cytoplasmic effects or interactions. The QTL at qSL.1@183 had a large effect corresponding to a ~ 3.5 cm increase in seedling length with a confidence interval of 3.5 Mbp and localized at the end of chromosome 1 (~ 38.4 Mbp). The second QTL, qSL.3@218, had a small confidence interval of ~ 1 Mbp but the effect size was moderate. The third QTL at qSL.5@160 had a very large confidence interval (~ 9 Mbp) with small effect size.

**Table 1:** Estimated QTL models: effect and localization.

Phenotypes	QTL model	Chromosome	Position	LOD	Lower CI	Upper CI	%Variance	%Variance (QTL^a^ Cyto)	Positive allele
SL	qSL.1@183	1	183.00	13.42	175.11	190.57	20.4	NA	Horkuch
SL	qSL.3@218	3	218.00	4.82	212.12	236.45	7.3	NA	Horkuch
SL	qSL.5@160	5	160.43	3.83	102.86	170.27	5.5	NA	Horkuch
RL	qRL.2@167	2	167.00	10.67	161.14	176.67	20.0	NA	Horkuch
TK	qTK.2@45 *Cyto	2	45.00	6.11	24.05	66.99	2.42	2.3	Horkuch^a^
TK	qTK.3@204 *Cyto	3	203.78	6.46	194.44	209.29	3.8	3.8	Horkuch^a^
PH	qPH.1@215	1	215.00	5.59	175.11	222.54	11.4	NA	Horkuch
PH	qPH.3@211	3	211.02	5.15	203.78	272.38	7.2	NA	Horkuch
PH	qPH.5@144 *Cyto	5	144.00	6.64	124.73	170.27	11.0	2.1	Horkuch^a^
ET	qET.7@97 *Cyto	7	97.00	5.82	85.83	104.41	17.7	5.5	Horkuch
FGN	qFGN.10@58 *Cyto	10	58.48	7.72	50.30	107.07	14.9	7.5	IR29
FGW	qFGW.10@58 *Cyto	10	58.48	9.13	50.30	107.07	17.6	9.1	IR29
SF	qSF.10@59 *Cyto	10	59.00	7.71	50.30	107.07	17.0	12.4	IR29
HI	qHI.10@104 + Cyto	10	103.75	8.48	50.30	107.07	15.4	1.4	IR29

Each QTL model was built by linear mixed model using kinship matrix as a covariate. Asterisk sign denotes interaction with cytoplasm whereas (+) sign denotes only additive cytoplasmic effect in the QTL model. ^a^Denotes QTL that has both main and interaction effect since only considering direction of the main effect can be misleading.

We identified one large effect QTL for RL at qRL.2@167 with a confidence interval of ~ 4 Mb. Here, the *Horkuch* parent contributed the positive allele. For TK, cytoplasm was a significant covariate that interacted with two QTLs which were detected at qTK.2@45 and qTK.3@203. In addition, the QTL qTK.2@45 is co-localized with the cluster of genetic loci that showed significant cytoplasm-nuclear association. The other QTL for TK, qTK.3@204 did not overlap with the association cluster in chromosome 3 but resided in close proximity. This evidence suggests a possible role of cytoplasmic-nuclear interaction for this trait.

#### QTL for reproductive stage treatment

**Figure 4:**
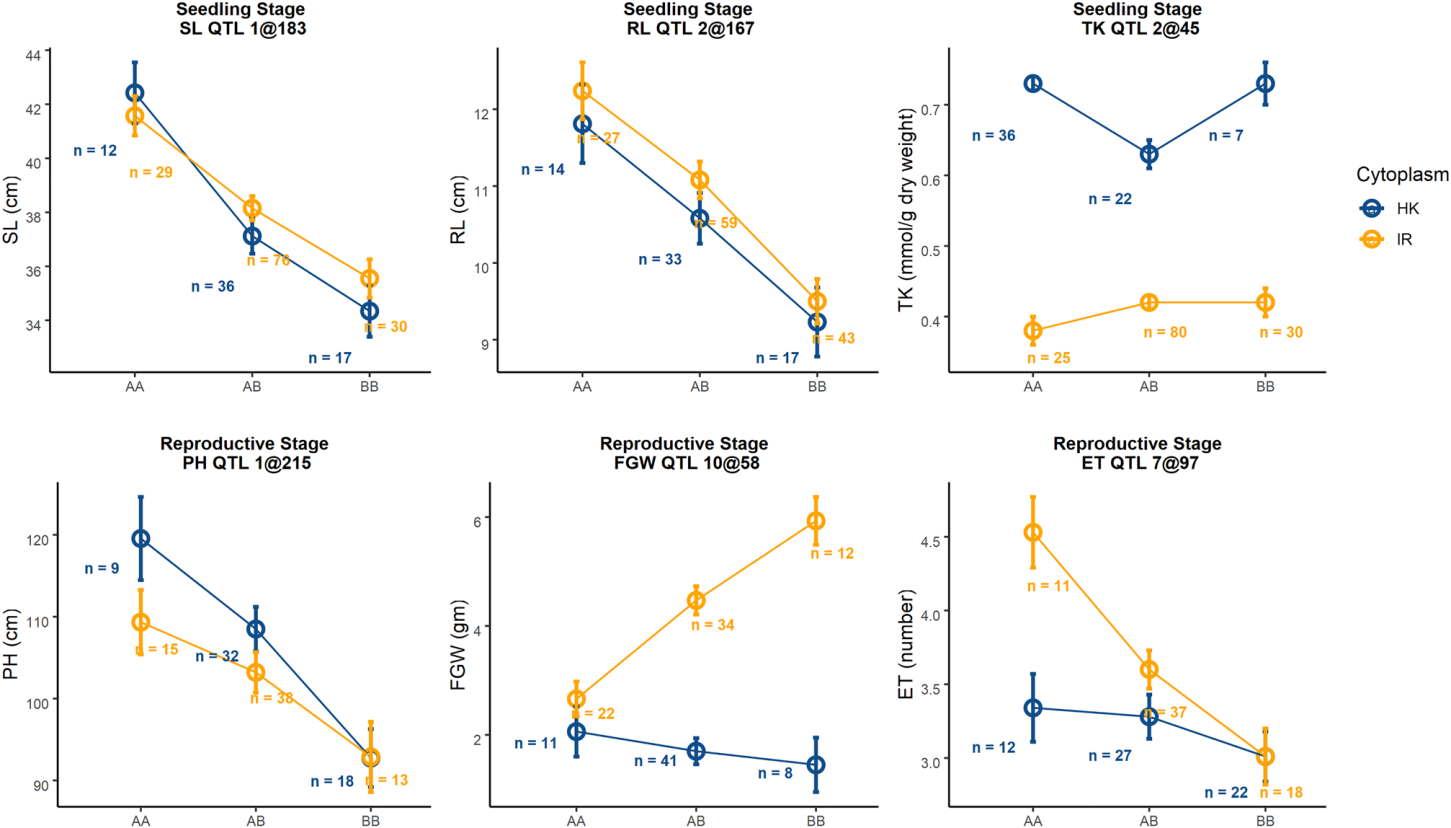
Interaction plots of allelic effect of QTL and cytoplasm on different traits from two different treatment stages. Blue line shows plants with *Horkuch* cytoplasm whereas orange line indicates plants with *IR29* cytoplasm. Alleles are plotted on x-axis where AA, AB and BB indicate homozygous *Horkuch*, heterozygous of *Horkuch*/*IR29* and homozygous *IR29* respectively. Allelic means ± SE are reported. Representative QTL effects for SL and PH are presented in the upper panel and exhibit no significant interaction with cytoplasm. The third plot from the left on upper panel demonstrates significant additive effects of the maternal cytoplasm on TK. In the bottom panel, plot two and three from the left demonstrate significant interaction of QTL alleles with cytoplasm for traits such FGW, ET.

For this stage, our primary focus was on yield responses of the plants under salt stress. We found 8 QTLs for 6 traits under salinity treatment at reproductive stage (Table 1, Fig. 3). A major effect QTL for FGN was found on Chromosome 10 at 58.5 cM (qFGN.10@58.5). Here, the *IR29* parent contributed the positive allele. We found significant cytoplasm-nuclear interaction for this QTL model where the *IR29* allele had positive effects only for *IR29*♀ (Fig. 4). However, this QTL had a very wide confidence interval of ~ 6 Mbp. We found two co-localized QTLs in this same region including a QTL for FGW and another for SF at qFGW.10@58.5 and qSF.10@59 respectively. These two QTLs also had significant interactions with cytoplasm in their corresponding QTL models. *IR29* contributed the positive allele for both qFGN.10@58.5 and qFGW.10@58.5. For FGW, as like FGN, the *IR29* allele had a positive effect only for *IR29*♀ but for SF this allele not only had positive effect for *IR29*♀ but also had a negative effect for *Horkuch*♀. We also found a QTL for HI at qHI.10@104 for which the positive allele was from IR29. However, this model only had an additive effect of cytoplasm.

Another QTL, qET.7@97 cM was detected for ET where the *Horkuch* parent contributed the positive allele. Similar with the other grain related traits, cytoplasm contributed significantly to this QTL model. However, the positive allele from *Horkuch* performed better in *IR29*♀. We also found three PH QTLs occurring at qPH.1@215, qPH.3@211 and qPH.5@144, for which the positive alleles were from the *Horkuch* parent. The first two QTL models showed only additive contribution of cytoplasm but the third showed also an interactive effect of cytoplasm. Overall, we found a hotspot of QTL on chromosome 10 for multiple parameters related to yield. The significant correlation of these traits may reflect related metrics of yield performance in rice.

### QTL co-localization

In this study, we found that some QTL intervals of various traits overlapped, therefore we annotated these overlapping intervals as QTL clusters. We detected a co-localized QTL at 1@175:220 cM [QTL Cluster 1 (QC 1)] affecting PH and SL from two different stages of treatment. These traits represent the vigor of plants at the two different growth stages and had significant positive correlation (Pearson’s correlation coefficient = 0.54). In parallel with this correlation, these two QTLs had positive alleles from the *Horkuch* parent and therefore may play a role in driving this correlation at the trait level. Another wide co-localized cluster was found at 3@194:273 cM [QTL Cluster 2 (QC 2)] impacting SL, PH and TK with the positive allele from the *Horkuch* parent. However, TK showed no significant correlation with PH which was causal for the co-localization for QC1. A third QTL Cluster 3 (QC 3) was found at 5@144:170 for SL and PH where the positive alleles were from *Horkuch* parent. The fourth QTL Cluster (QC 4) at 10@58:107 was found for four yield related traits including FGN, FGW, SF and HI for which the positive alleles were from the *IR29* parent.

In an earlier study^47^ the same mapping population had been implemented to examine the genetic basis of salinity response with a different QTL analysis framework in which the genetic map had been constructed by using Double digest restriction site-associated DNA (ddRADseq) technology. However, that genetic map failed to represent more than half of the genomic space due to erroneous genotyping and high rate of missing SNP calls. Nevertheless, in that study several QTLs for the traits studied in this current work had been detected. In order to compare the QTLs identified in these two studies we tested for overlaps between QTL intervals for a given trait (SL, RL, TNa, TK, PH, ET, FGN, FGW, or HI) and categorized QTL as: Category 1: found in both studies, Category 2: found in both studies by relaxing the threshold of type-I error for this current study, Category 3: found exclusively in this study, and Category 4: found exclusively in Noor et al.^47^. For category 1 we could not detect any QTL which overlap between these two studies without lowering the threshold for type-I error. However, in category 2 we detected one QTL peak each for TK and TNa on chromosome 10 at ~ 18 Mbp while lowering the permutation threshold in our study. In category 3, there are 14 QTLs (Supplementary Table S6) for which the QTL intervals span the genetic space which was uncaptured in the earlier study. We detected 5 QTLs in category 4 which were clusters in one single hotspot of the earlier genetic map. This hotspot was at chromosome 11 which span ~ 18 Mbp to ~ 19 Mbp genomic region and careful investigation of the LOD profile revealed that one single marker was the causal reason for high LOD peaks for the QTL detected at that given interval for majority of the traits which were highly correlated with each other (TNa, TK, FGW, FGN and HI).

### GO enrichment analysis of candidate genes within QTL confidence intervals

To understand the molecular mechanism of salt tolerance, we further investigated the function of candidate genes that were located within the QTL confidence intervals (Supplementary Table S3). We applied GO enrichment analysis on the candidate gene lists against the genome-wide background frequency of GOs. The results for GO enrichment analysis have been provided in Supplementary Table S4. For the seedling stage, the QTL at qSL.1@183 showed significant enrichment of GO terms such as pollination, protein lipidation, lipoprotein and liposaccharide metabolic process, various transport and DNA-directed RNA polymerase complexes. Another QTL for SL at qSL.3@218 was significantly enriched with the GO terms such as protein transport and localization, amide and lipid transport, cytoskeleton and actin binding. The third QTL for SL, qSL.5@160 had significant enriched GO terms for chromatin assembly, nucleosome organization, DNA packaging, ion transport, amide and peptide transport. QTL qRL.2@167 showed significant enrichment of GO terms such as carboxylic, dicarboxylic and C4-dicarboxylate transport, malate transport, anion transport and sexual reproduction. Two QTLs for TK, qTK2@45 and qTK3@204, were both enriched with the GO terms oxidoreductase activity, various transmembrane transporter activity, potassium ion transmembrane transporter and cation transmembrane transporter.

For traits at reproductive stage, the QTL qPH.1@215 was significantly enriched with the GO terms anion and potassium ion transmembrane transport, divalent metal ion transport, pollination, reproduction process, endoplasmic reticulum and organelle sub-compartment. Another PH QTL, qPH.3@211 showed enrichment for GO terms such as cellular nitrogen compound metabolism, organic acid transport, mitochondrial membrane and protein complex. The third QTL, qPH.5@144 also showed significant enrichment for nitrogen compound metabolic process. This QTL was also enriched for various mitochondria and cytoplasm related GO terms. The QTL, qET.7@97 was enriched with cytoplasm and mitochondrial membrane part and chlorophyll metabolic process. QTLs for FGW, FGN, SF and HI share the same QTL intervals therefore the gene models within this interval were identical. This interval was enriched with cell wall macromolecule catabolic process, amino sugar and glycan metabolic process, protein localization to organelles and mitochondrial transport. The significant enrichment of mitochondrial and organelle related GO terms for some QTL confidence intervals suggests a possible explanation for the significant cytoplasm and cytoplasmic-nuclear interactions detected in our study.

For QTL models that had significant cytoplasmic effect, 1473 annotated genes are present in the respective confidence intervals. In our previous studies we tested for the association of gene expression to salinity stress for this same reciprocal mapping population at seedling and reproductive developmental stages^56,57^. Among the genes that are present in the QTL confidence interval, 188 showed significant cytoplasm × treatment interaction in these previous gene expression studies. In order to identify their association for cellular component we further tested for enrichment of GOs for these common genes that are present in QTL intervals and showed significant cytoplasm × treatment interaction in our previous gene expression studies (Supplementary Table S5). We found these genes to be significantly enriched with GO terms such as mitochondrial proton-transporting, ATP-synthesis complex, mitochondrial protein-complex and mitochondrial membrane.

## Discussion

In this study, we explored the responses of rice to salinity stress at two different growth stages using a reciprocal mapping population. Among the 14 QTLs that we discovered, 8 QTLs models showed significant effect of cytoplasm. This finding underlines the importance of considering both organelle and nuclear genome for complex traits such as salinity tolerance.

Cytoplasmic background may play an important role in trait genetic architecture by itself or through complex interactions with the nuclear genome^58–61^. Gregorio and Senadhira^25^ reported significant reciprocal effects among nine different crosses for salinity response in rice varieties and suggested using the susceptible plants as the male parent. In order to identify the best candidates for QTL pyramiding by breeders, it is essential to accurately estimate individual QTL effects for the trait of interest. Hence, it is important to test for the effect of covariates such as cytoplasm in a QTL model and estimate its effect size. In this way, the causality, contribution and combinations of cytoplasm and nuclear-donor alleles of QTL can be defined. Moreover, including potential predictors as covariate in QTL mapping can increase the ability to detect small-effect QTL peaks if there is a significant contribution of that predictor for that given trait. Considering these aspects, we employed a QTL modeling framework where the cytoplasm-nuclear interaction was also considered as a contributor to phenotypic variance. For TK, FGN and FGW, the additive effect of cytoplasm was significantly large compared to the effect of a single QTL (Fig. 4). Identification of causal impacts of cytoplasm will help to define the best combination of cytoplasm and nuclear-donor materials and will underscore the selection trade-off for multiple desired traits. For instance, on the one hand, we found the positive nuclear allele of *IR29* had its effect only in *IR29*♀ for the QTL model of yield related traits; while on the other hand we observed the strong positive effect of *Horkuch* cytoplasm for the QTL model of TK at the seedling stage. The latter trait is a highly desired one for breeding of salt tolerant varieties. Hence, estimating the contributions of cytoplasm for multiple traits can help understand the performance trade-off in a breeding program for QTL pyramiding.

The cytoplasmic genome can influence interaction of alleles from nucleus and cytoplasm and can favor the evolutionary co-adaptation of high-fitness. We found a significant association of cytoplasm for some traits and therefore further tested for non-random interaction of alleles for nucleus and cytoplasm. We found that the QTL qTK.2@45 was a hotspot of cytoplasm-nuclear interaction on chromosome 2. Additionally, qPH.5@144 was another similar hotspot on chromosome 5. Both of these QTL models showed significant effect for cytoplasm. For qTK2@45, the effect of cytoplasm was mostly additive where *Horkuch*♀ contributed a large positive effect. On the contrary, for qPH5@144, cytoplasm had an interactive effect. *IR29* nuclear allele had a negative effect on PH and homozygous *IR29* nuclear allele on *Horkuch*♀ had even larger negative effect size (Supplementary Fig. S3). This suggests a significant interaction of nuclear alleles with the cytoplasmic genome. This further supports the fact that selection of the female plant plays an important role for the performance of a breeding population and while pyramiding QTL and the conditional selection of cytoplasm may have some trade-off on a hybrid plant’s performance. Moreover, we detected significant cytoplasm-nuclear linkage of a few markers that overlapped with some QTL intervals. Therefore, careful consideration is needed in order to select these loci for QTL pyramiding.

One important finding in this study is that we have detected multiple co-localized QTLs within and among the two different stages of salinity treatment. This finding emphasizes the possible constraints during selection of QTL pyramiding in a breeding program. We identified four QTLs clusters where multiple trait QTL co-localized. The QTL for PH and SL are colocalized at QTL cluster 1 on chromosome 1. This cluster had a positive effect for the *Horkuch* parental allele for PH and SL. However, a taller plant is not the desired plant architecture for a breeding program for high-yielding rice varieties since this will lead to over-investment of energy in vegetative growth and potential lodging. On the other hand, Leon et al.^62^ reported that percent of shoot length reduction under saline treatment is highly correlated to saline sensitivity. This conditional relationship between traits results in some possible trade-offs between favorable and undesirable traits. The same logic is applicable for QTL cluster 2 where traits (SL, TK and PH) for these clusters are positively correlated but increased PH is not desirable. On the other hand, for QTL cluster 4, all the yield related such as FGN, FGW, SF and HI could be combined where the *IR29* parent contributes all the positive alleles. FGN, FGW, and SF are highly correlated traits i.e. characteristics of the rice seed number and weight, hence the data found overlapping QTLs. Taken together, these findings underscore the importance of studying the performance of a plant for different developmental stages. In addition to that, we need to consider the fact that selection for multiple traits may not be orthogonal due to the complex mechanisms of salt adaptation.

To understand the molecular mechanism of salt response and the effect of cytoplasm for salt tolerance we tested for enrichment of GO functions for genes within QTL confidence intervals. Both the QTL intervals for TK were enriched with various transmembrane transporter activity, and potassium ion transmembrane transporters. K^+^ is involved in numerous metabolic processes in plants and excess Na^+^ interferes with the K^+^ homeostasis during salinity stress^63^. In the qTK3@204 interval, a specific peroxidase was detected as a cis expression QTL (Seraj et al., Unpublished data). Peroxidases normally reduce reactive oxygen species under stress and can contribute to regulation of HAK type potassium transporters. Also, in the qTK2@45 region a calcium transporting ATPase was detected as a cis eQTL that showed differential expression in the two parents (Seraj et al., unpublished data). This is a less characterized plasma membrane calcium ATPase in rice named OsACA2^64^ that catalyzes the hydrolysis of ATP coupled with the transport of calcium in cytosol and maintains calcium homeostasis under salt stress Normally under salt stress an increase in Ca^2+^ ensues in a time-sensitive manner and the homeostasis is maintained by Ca^2+^ channels, Ca^2+^ exchangers and Ca^2+^ ATPases^65^. Also, calcium signaling pathways have an important role in activation of potassium channels needed to maintain potassium homeostasis under salt stress.

For the reproductive stage, we found most of the QTL intervals for PH, ET, FGW, FGN, SF and HI were enriched with mitochondria, cytoplasm and organelle related GOs. This supported the observation that these QTL models also showed significant interaction with cytoplasm. Additionally, enrichment analysis of DEGs (significant for cytoplasm × treatment model) from our previous studies^56,57^ on this mapping population were enriched with GO terms such as organelle, thylakoid, mitochondria, photosynthesis, cation transmembrane transporter and various sodium symporter activities. Salt stress inhibits photosynthesis of plants but how this affects the ionic balance of chloroplasts has not been studied much, until recently. Bose et al.^66^ has proposed some candidate transporters that are involved in the movement of sodium, potassium and chloride across chloroplast membrane in glycophytes and halophytes and explained how these transporters may regulate photosynthesis in chloroplast. These candidate symporters include bile acid: sodium symporter and cation transmembrane transporter which have possible roles in maintaining chloroplast ion homeostasis. From our gene expression studies of cytoplasm × treatment DEGs, enrichment of symporter GOs that are localized in mitochondria and organelles suggest a possible role of mitochondria and chloroplast during salinity stress and tolerance. The bile acid: sodium symporter gene also appeared as a trans expression QTL under salt stress linked with the potassium QTL region qTK2@45 as well as qPH.3@211 (Seraj et al., unpublished data). This evidence also suggests a plausible explanation why we found cytoplasm as a covariate in QTL models for this study. These are likely candidates for future functional genomic studies of salinity tolerance.

In this QTL analysis framework, we applied linear mixed model which can handle cytoplasm and alleles as fixed effect predictors. We also included kinship as a covariate to reduce background polygenic variation except for the chromosome that is being tested to minimize the over estimation of QTL effect size. In our previous study, the genetic map generated by ddRAD technique failed to capture a significant space of genetic map due to erroneous genotyping and high rate of missing SNP calls^47^. In this current study, we implemented a robust QTL analysis framework on this improved genetic map and we were able to detect three QTLs for SL and one RL at seedling stage salinity treatment not detected earlier (Supplementary Table S6; Supplementary Fig. S4). For reproductive stage salinity treatment, we were able to detect additional five QTL for PH, ET and SF. We also detected one big effect QTL for FGN and FGW in a different chromosome that our previous study failed to capture due to missing markers at that region. In addition to that, this framework provided QTL with higher likelihood and tighter confidence interval and provided better estimation of effect size of each QTL for a given trait. Therefore, these robust QTL may contribute significantly for development of rice which combines both salt tolerance and high yields.

## Conclusion

In this study, we aimed to identify genetic loci for salinity tolerance of a rice landrace, *Horkuch,* at two sensitive developmental stages. We found 14 QTLs for 9 traits under salinity treatment. We detected some overlap in the genomic regions affecting traits across developmental stages. One chief finding of this study was the significant contribution of cytoplasm on many traits and its effect on the corresponding QTL model. Enrichment analyses suggest that the observed cytoplasmic effect could be causally related to plastid symporter activity and their interaction with nuclear genes. Collectively, this study helped to understand the genetic basis of salt tolerance mechanisms of a local rice landrace *Horkuch*. Moreover, careful implementation of pyramiding of QTLs that were detected in this study will help to generate high yielding salt tolerant rice varieties.

## Supplementary Information

Supplementary Information 1.

Supplementary Information 2.

Supplementary Information 3.

Supplementary Information 4.

Supplementary Information 5.

## Data Availability

The datasets analyzed during the current study are included within the article except the genetic map and phenotypes which are available in Dryad repository (https://datadryad.org/stash/share/DFLm7PqkWv17OgFgBCWcio21jkcOgGEL7VDaiXaHUaI).

## Abbreviations

QTL: Quantitative trait loci
SES: Standard evaluation system
Tchlr: Total chlorophyll content
SL: Shoot length
RL: Root length
TNa: Total sodium
TK: Total potassium
K/Na: Potassium by sodium
PE: Panicle exsertion
TT: Total tiller number
ET: Effective tiller number
PH: Plant height
HI: Harvest index
FGW: Filled grain weight
FGN: Filled grain number
SF: Spikelet fertility
SNP: Single nucleotide polymorphism
QC: QTL cluster
GO: Gene ontology
