# Supplementary material for: Salt tolerance QTLs of an endemic rice landrace, Horkuch at seedling and reproductive stages
Source: Sci Rep. 2022 Oct 15;12:17306. doi: 10.1038/s41598-022-21737-9 (PMC9569374; doi:10.1038/s41598-022-21737-9)
Supplement: Supplementary file 1 — Supplementary Information 1. [file 41598_2022_21737_MOESM1_ESM.docx]

**Supplementary Information**

**Figure S1.** A. Genetic map of this F2 mapping population where each vertical line segment represents one chromosome and horizontal line segment of each chromosome represent the position of genetic marker in CM. B. Plot of physical vs genetic distance of markers for the linkage map.

**Figure S2.** Interaction plots of allelic effect of QTL and cytoplasm at seedling stage salinity treatment. Blue line shows plants with Horkuch cytoplasm whereas orange line indicates plants with IR29 cytoplasm. Alleles are plotted on x-axis where AA, AB and BB indicate homozygous Horkuch, heterozygous of Horkuch/IR29 and homozygous IR29 respectively. Allelic means +/- SE are reported.

**Figure S3.** Interaction plots of allelic effect of QTL and cytoplasm at reproductive stage salinity treatment. Blue line shows plants with Horkuch cytoplasm whereas orange line indicates plants with IR29 cytoplasm. Alleles are plotted on x-axis where AA, AB and BB indicate homozygous Horkuch, heterozygous of Horkuch/IR29 and homozygous IR29 respectively. Allelic means +/- SE are reported.

**Figure S4.** Comparison of two different QTL models for TK and FWG. Estimated LOD score for each model were plotted against the corresponding physical position of true markers. Orange points represent the LOD profile for new linkage map built using DArtSeq technique while blue points represent the old linkage map constructed from ddRAD data. Chromosomes that had one significant QTL in either model have been plotted.

**Table S1.** Descriptive statistics of phenotypes measured at seedling and reproductive stages under salinity stress

**Table S2.** Detailed statistics for QTL

**Table S3.** List of genes in QTL confidence interval where one sheet represents one single QTL

**Table S4.** List of significant GO terms for genes in QTL confidence interval where one sheet represents one single QTL

**Table S5.** List of significant GO terms for the common genes between all candidate genes of QTL confidence interval and significant DEGs for cytoplasm*treatment interaction model of seedling shoot tissue, seedling root tissue and reproductive shoot tissue

**Table S6.** Comparison of detected QTL in current study vs. previous study

**
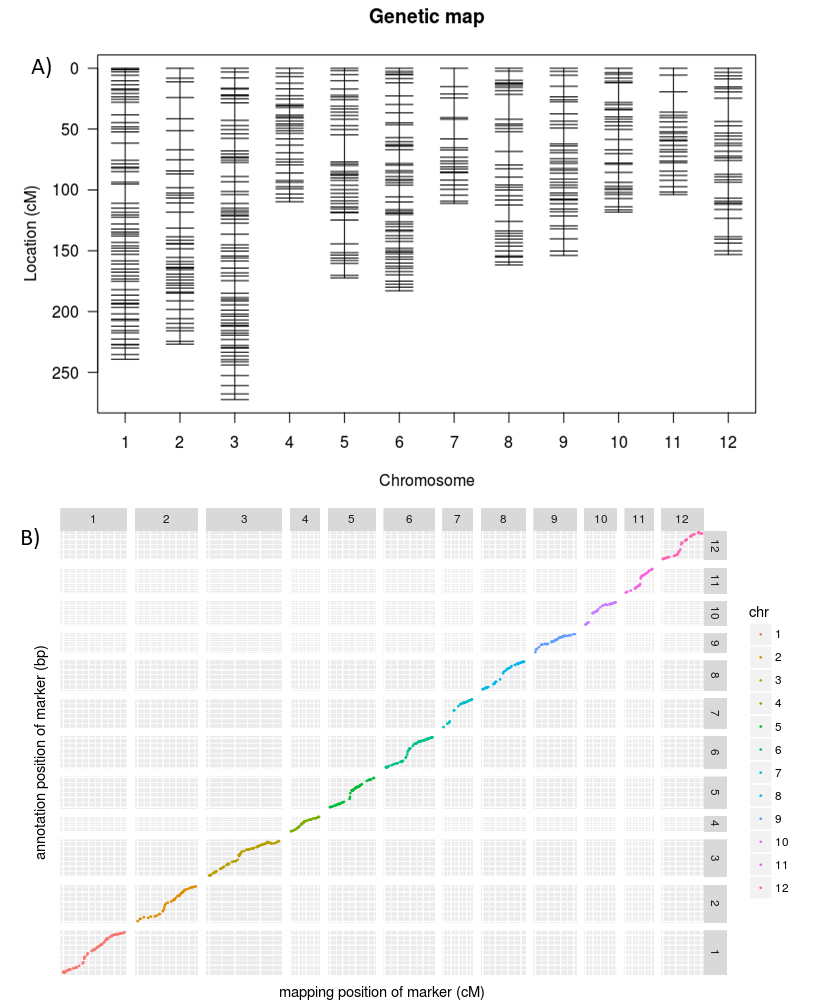
**

**Figure S1.** A. Genetic map of this F_2_ mapping population where each vertical line segment represents one chromosome and horizontal line segment of each chromosome represent the position of genetic marker in CM. B. Plot of physical vs genetic distance of markers for the linkage map.

**
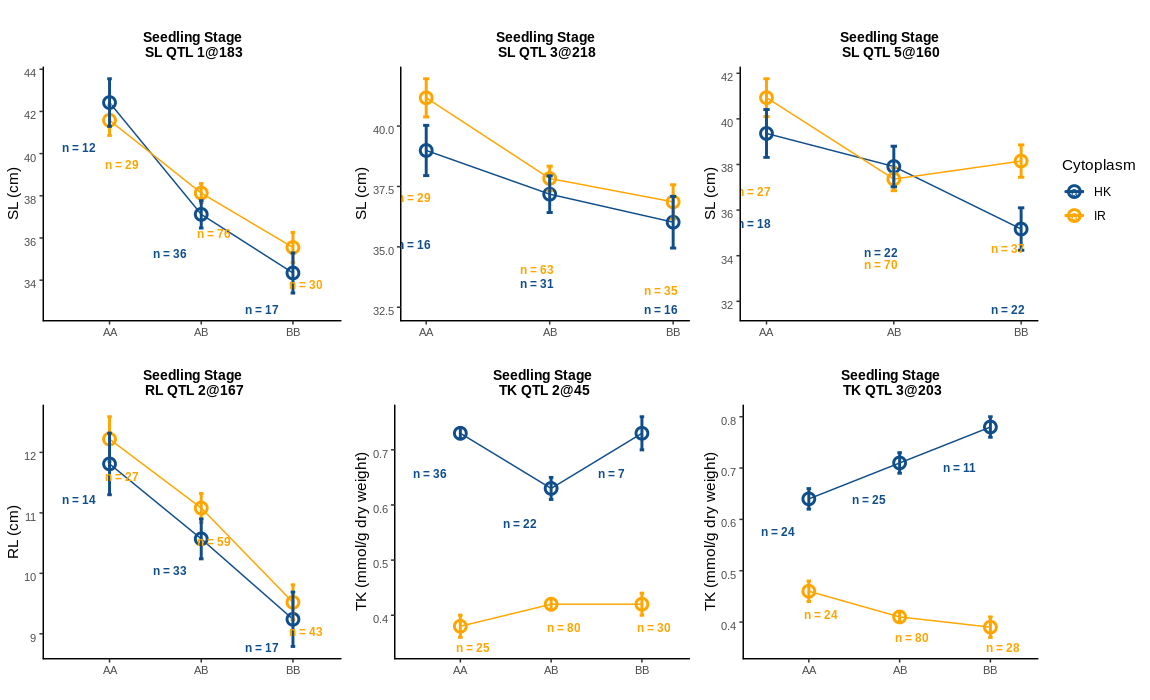
**

**Figure S2**. Interaction plots of allelic effect of QTL and cytoplasm at seedling stage salinity treatment. Blue line shows plants with Horkuch cytoplasm whereas orange line indicates plants with IR29 cytoplasm. Alleles are plotted on x-axis where AA, AB and BB indicate homozygous Horkuch, heterozygous of Horkuch/IR29 and homozygous IR29 respectively. Allelic means +/- SE are reported.


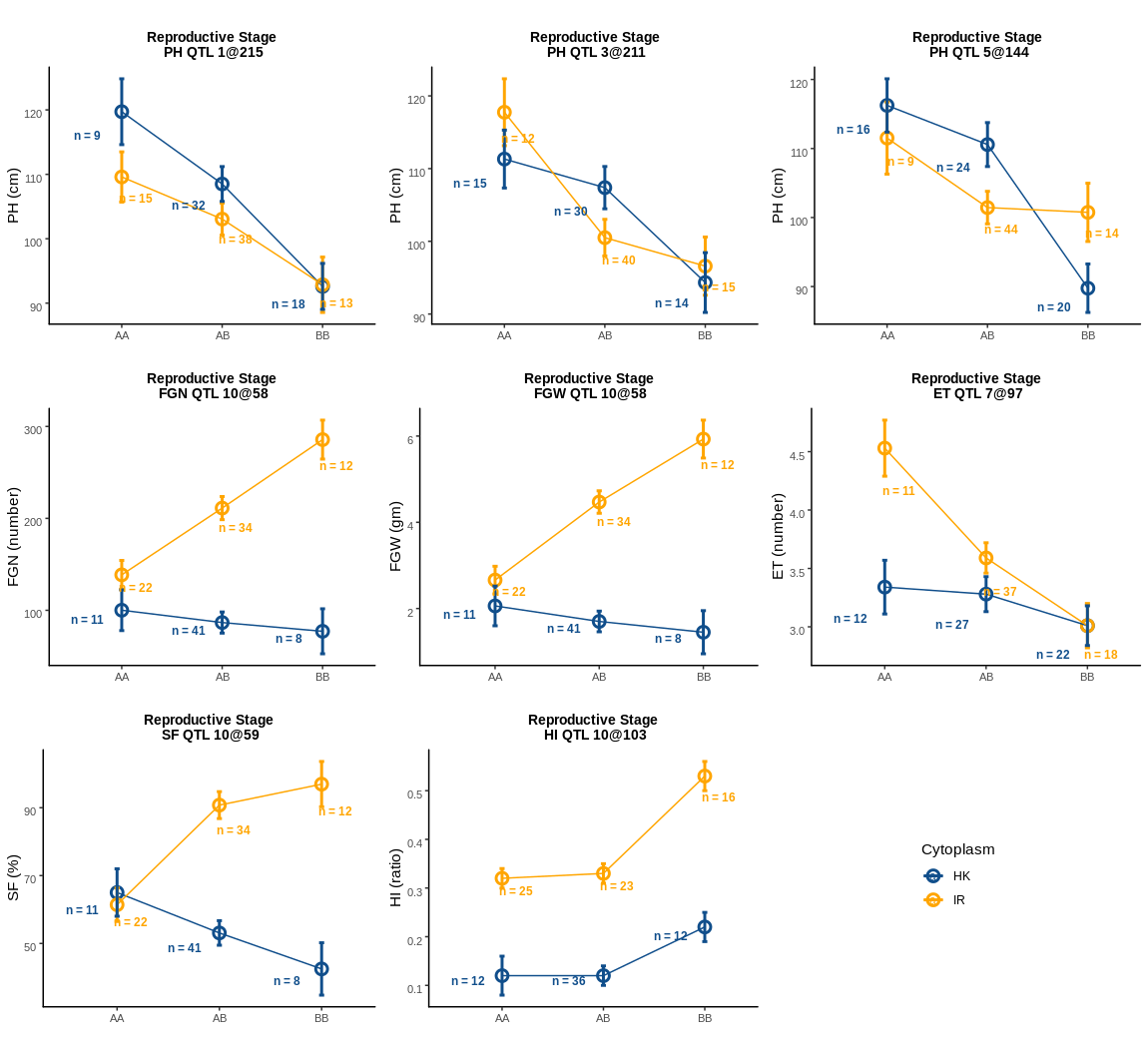


**Figure S3.** Interaction plots of allelic effect of QTL and cytoplasm at reproductive stage salinity treatment. Blue line shows plants with *Horkuch* cytoplasm whereas orange line indicates plants with *IR29* cytoplasm. Alleles are plotted on x-axis where AA, AB and BB indicate homozygous *Horkuch*, heterozygous of *Horkuch*/*IR29* and homozygous *IR29* respectively. Allelic means +/- SE are reported.


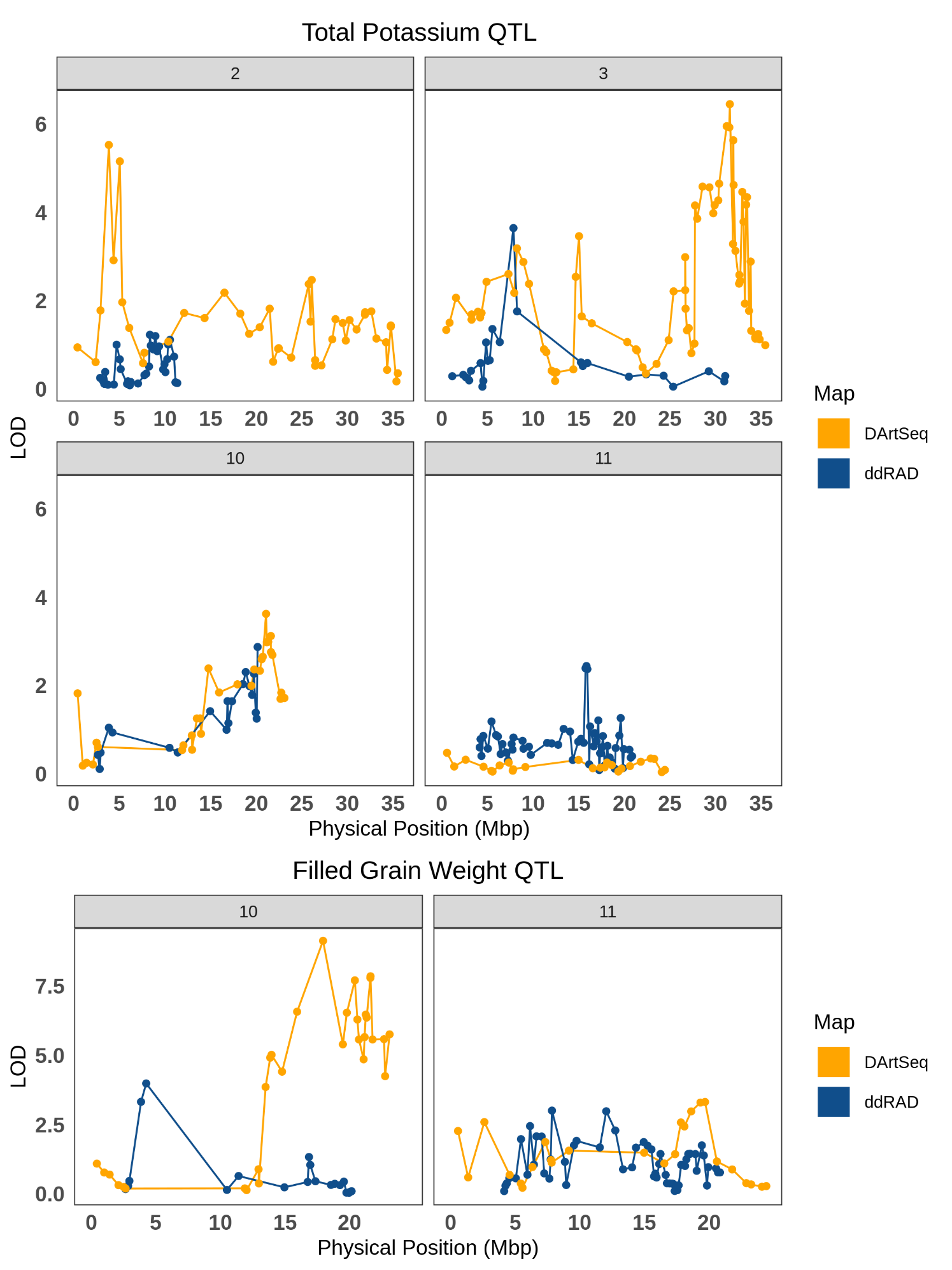


**Figure S4:** Comparison of two different QTL models for TK and FWG. Estimated LOD score for each model were plotted against the corresponding physical position of true markers. Orange points represent the LOD profile for new linkage map built using DArtSeq technique while blue points represent the old linkage map constructed from ddRAD data. Chromosomes that had one significant QTL in either model have been plotted.
